# Retraction of: “SCARA5 inhibits oral squamous cell carcinoma via inactivating the STAT3 and PI3K/AKT signaling pathways”

**DOI:** 10.1515/med-2025-9992

**Published:** 2025-10-24

**Authors:** Juan Huang, Chunhua Lv, Baoyu Zhao, Zhongqian Ji, Zhenran Gao

**Affiliations:** Department of Stomatology, Taizhou People’s Hospital, Tauzhou, 225300, China; Department of Stomatology, Taizhou People’s Hospital, No. 366 Taihu Road, Tauzhou, 225300, China

Retraction of: Huang J, Lv C, Zhao B, Ji Z, Gao Z. SCARA5 inhibits oral squamous cell carcinoma via inactivating the STAT3 and PI3K/AKT signaling pathways. Open Medicine 2023; 18(1): 0230627, DOI: 10.1515/med-2023-0627.

The retraction has been agreed upon at the request of the corresponding author, Dr. Zhenran Gao, with the consent of all co-authors, the Editor-in-Chief, Professor Vittorio Calabrese, and the publisher, Walter de Gruyter GmbH. The retraction is due to plagiarism of the plots included in Figure 3e and g, which were copied from 10.1002/iid3.785 and 10.3892/etm.2021.10711. In addition, the authors have acknowledged that there are discrepancies between the presented data and the conclusions of the article, which raises questions about the integrity and scientific rigor of the research.

